# Effectiveness of Brainball program on physical fitness of primary school pupils in Vietnam. A longitudinal study

**DOI:** 10.3389/fpubh.2023.978479

**Published:** 2023-01-26

**Authors:** Van Han Pham, Andrzej Rokita, Ireneusz Cichy, Sara Wawrzyniak, Michał Bronikowski

**Affiliations:** ^1^Department of Team Sports Games, Wrocław University of Heath and Sport Sciences, Wrocław, Poland; ^2^Department of Physical Education, An Giang University, Vietnam National University (VNU)-Ho Chi Minh, Ho Chi Minh City, An Giang, Vietnam; ^3^Department of Didactics of Physical Activity, Poznan University of Physical Education, Poznań, Poland

**Keywords:** Brainball, educational balls, physical education, 2nd-grade students, integrated teaching

## Abstract

**Objective:**

The present study aimed to evaluate the effectiveness of the Brainballs on the physical fitness of 2nd-grade students at a primary school in Vietnam during and eight months after the experiment.

**Methods:**

The study included 55 pupils (23 boys and 32 girls) aged seven years. The study design was a pedagogical experiment with a parallel-group technique, including experimental and control groups. The examination was carried out in 2019/2020 in three terms pre- (September 2019), post- (January 2020), and follow-up (September 2020). Physical fitness was tested with the use of the International Physical Fitness Test. The Brainball program, conducted twice a week for 35 minutes, combined physical education (PE) with subject-related content, utilizing 100 balls with painted letters, numbers, and signs.

**Results:**

Results show that the fitness level was not increased significantly after 20 weeks of the intervention program, neither in experimental nor control groups. However, it significantly improved eight months later at the follow-up examination. The analysis of covariance indicated that pupils from the experimental group improved significantly on most physical fitness as compared to the control group, specifically on the following tests: 50-meter running (*p* = 0.0044), toe touch (*p* = 0.0137), standing long jump (*p* = 0.0076), 4 × 10 m sprint (*p* = 0.0333), hand strength (*p* = 0.0233).

**Conclusions:**

These results have shown long-term positive effects of the use of “Brainball” educational balls in physical education classes on the physical fitness development of students, especially in the qualities of speed, strength, and flexibility.

## 1. Introduction

Evidence (1) proves that physical activity (PA) plays a fundamental role in stimulating a sound development of a growing child. It supports optimal biological growth, physical development, and psychological health (1). Researchers and education experts agree that PA and spontaneous play remain essential in a child's overall development (2). The PA in childhood may have numerous benefits on health, brain and emotional functioning (3, 4). Findings from research (5–7) indicate that PA if taken regularly, enhances the strength of bones and muscles. It also improves the cardiovascular system with better blood circulation, thus reducing the risk of chronic diseases such as (obesity, cancer, cardiovascular disease, diabetes, and metabolic syndrome) (7). In addition, the World Health Organization (WHO) recommends that children and adolescents aged 5–17 should take at least 60 min of moderate to vigorous-intensity PA daily to improve cardiorespiratory and muscular fitness, bone health and reduce symptoms of anxiety and depression (8).

For children and adolescents, PA includes games, sports, recreation, physical education (PE), or planned exercise as part of the family, school, and community activities (8). However, recent research shows that schools are ideal environments to encourage long-term regular physical activity (9, 10) and that physical education provides students with the opportunity to engage in enjoyable physical activity, become physically fit, and learn general motor and behavioral skills (11). The importance of physical education in child and adolescent development has been demonstrated by many researchers around the world (9, 10, 12, 13). These authors agree that an appropriate physical education program that focuses on fun games and exercise can help students have more significant opportunities to participate in physical activity and has many physical and mental health benefits. In a study aimed at determining the effectiveness of using Ringo games in physical education classes (14), the authors showed that after an experimental semester, students in the game-based program demonstrated higher fitness than those in the traditional exercise group program. Other research, in Italy (15), interwoven 2 h of intervention scheduled into regular school curricula, where the contents was designed and supervised by professional PE experts. The findings of the study after 8 months of implementation indicated that studying PE in the school settings can be very helpful with school-aged children to foster their physical, mental, and psychological health.

In an effort to find a new teaching method to improve teaching and learning for students, researchers at the Wroclaw University of Health and Sports Science, Poland, have created an active teaching method called Eduball/Brainball. The primary teaching method of the program is to use games and exercises designed with educational balls to integrate the contents of other subjects into physical education classes (16, 17). After years of research, researchers have proven that using fun games and exercises along with educational balls in physical education classes has positively impacted children's physical fitness (16, 18), motor skills (19–22), eye-hand coordination, and spatial awareness (23–26). Children who participated in pedagogical experiments with Eduball significantly improved their language skills and achieved better math scores than children in the control group (16, 27–29).

The idea of the educational balls was coined in 2002 in the Department of Team Sports Games at the Wroclaw University of Health and Sport Sciences, Poland. The first name of educational balls was “Edubal.” After 10 years of experience and research with Edubals, the set was modified and the next edition of educational balls, called “Eduball,” was prepared. In 2018, the English edition of education balls called “Brainball” started; although the name is different, the idea of Edubal/Eduball/Brainball is the same; children learn while playing! (30).

Researchers have found that physical activities with balls are the favorite forms for children. They designed the educational balls by adding numbers, letters, and mathematical symbols on the balls' surface and adjusting the balls' size to fit the children's body size (from 6 to 9 years old) (31). The set of Brainball consists of 100 balls for mini team sports games (basketball and soccer) in five colors (yellow, green, blue, red, and orange) with painted (black) letters of the alphabet (uppercase and lowercase letters); numbers (from 0 to 9); and signs of mathematical operations [addition (+), subtraction (–), multiplication (^*^), division (:), greater than (>), less than (<), parentheses (), and the at sign (@)] (30). The numbers, letters, signs, and colors of the educational balls make their extensive use possible in almost all school subjects. They can be used to teach native languages (Polish or English), foreign languages (English or Spanish), mathematics, history, biology, geography, etc. (30). The games and exercises of Brainball are based on natural forms of movement (running, jumping, throwing, catching, etc.) combined with physical activity so that students can easily acquire and improve basic motor skills and develop physical and academic fitness achievements (27, 32).

Eduball/Brainball has been implemented in the official list of teaching aids for elementary schools in Poland. They were accredited and approved by the Polish National Ministry of Education. Eduball/Brainball has also been launched and confirmed its benefits in Germany, Portugal, Finland, Greece, the USA, Singapore, and Taiwan (China) (28). However, there are no scientific studies evaluating Eduball/Brainball program in Vietnam. This study aimed to investigate whether teaching PE with Brainball can have similar impact and significance on the physical fitness levels of elementary pupils in Vietnam.

## 2. Methods

### 2.1. Participants

The research sample was students from two second-grade classes in a primary school in the center of An Giang province, a province located in the Mekong Delta region of Southern Vietnam. A total of 55 students (23 boys and 32 girls, ages: 7) participated in the study. The study was conducted during the 2019–2020 academic year. The study design included pedagogical experiments using parallel grouping techniques under natural conditions. Participants were randomly divided into a control group of 27 students (11 boys and 16 girls) and an experimental group of 28 students (12 boys and 16 girls). The teaching process of both groups (experimental group and control group) was conducted according to the same curriculum prescribed by the Ministry of Education and Training of Vietnam. The only difference is the introduction of Brainball into the teaching and learning in the experimental group. All participants' parents and guardians signed informed consent for their children to participate in this study. The study was approved by the local Ethics Committee for Research Involving Human Subjects (Senate Committee on Ethics of Scientific Research at the Wroclaw University of Health and Sport Sciences on September 22, 2010). It was conducted according to the principles of the Declaration of Helsinki.

The experimental factor was a PE program combined with Brainball games and exercises (Table 1). The experimental group participated in physical education classes twice a week for 35 min each and integrated with Brainballs for 5 months (one semester). Each class includes warm-up periods (5 min), Brainball games and exercises (15 min), physical education lessons (10 min), and cool down (5 min). The teacher guides the students to perform movement exercises at the warm-up stage. At the Brainball games and exercises stage, the teacher teaches students to play 2–3 games or exercises. Most games and activities involve movement, such as running, jumping, throwing, catching, etc.

**Table 1:** Physical education of experimental and control groups.

**Experimental group**	**Control group**
- Warm up	- Warm up
- PE unit	- PE unit
- Brainball games and exercises	- Drills
- Cool down	- Cool down

In the control group, physical education classes also took place twice a week for 35 min and were conducted using traditional styles (without Brainballs). The same PE teacher with 10 years of experience, taught physical education in both groups (experimental group and control group). Due to the experiment the teacher was trained in the Brainball method to organize and perform games and exercises with these balls.

### 2.2. Research tool

The International Physical Fitness Test (33) was used to assess the physical fitness level of students. Seven of the eight tests of the International Fitness Test were performed, including the 50-meter sprint, standing long jump, hand strength, bent arm hang, 4 × 10 m sprint, sit-ups, and forward bend. The 600 m sprint test was rejected for lack of parental consent.

#### 2.2.1. 50-meter sprint

At the command “on your marks,” the pupil doing the exercise stands still in front of the starting line with one leg put forward (a so-called standing start). Then, at the “start” signal, he runs to the finish as quickly as possible. Time is measured with an accuracy of 0.1 s.

#### 2.2.2. Standing long jump

The subject stands with legs extended; naturally, toes are placed close to the boundary line; when jumping and landing, both feet do it simultaneously. The jump length is measured from the setline (beam) to the nearest footstep left by the jumper's heel. The result is the longest distance jumped, the best of two attempts.

#### 2.2.3. Hand strength

The subject stands with feet shoulder-width apart. The dominant hand holds the dynamometer toward the palm and extends straight along the body. The subject performed the exercise by reducing the dynamometer to maximum power. The result is the highest of two attempts.

#### 2.2.4. Bent-arm hang

The task is to remain as long as possible, hanging with arms bent in elbow joints. Upon starting the test, the person doing the exercise holds the bar with fingers directed downwards and the thumb from the bottom upwards, at the shoulders' breadth, so that his chin would be above the bar. The test starts when the person doing the exercise hangs on the bar unaided and ends when his eyes go below the bar. Time is measured in 0.1-s units.

#### 2.2.5. Sit-ups

The subject lies supine on a mattress, knees bent, hands clasped on the neck, and performs sit-ups, feet held firmly by an assistant. The result is the exact number of sit-ups in 30 s.

#### 2.2.6. 4 × 10 m sprint

The subject stands with one foot forward (standing start) in front of the starting line. On signal, the subject runs to the finish line to pick up a block, runs back, and places the block behind the start line. The subject then runs back to the finish line, picks up a second block, and runs back to put it behind the start line. If the block is thrown and not placed behind the starting line, the test is considered invalid and must be repeated. Time is measured with an accuracy of 0.1 s.

#### 2.2.7. Forward bend

The subject was not wearing shoes, stood on a stool or bench, toes placed close to the edge of the stool, feet together, and knees straight. From this position, the person doing the exercise bends forward with a continuous movement to reach the furthest with his fingers. Such a position of a maximum bend must be kept for 2 s. If the person doing the exercise reaches the level he is standing on while bending with a continuous movement, he scores 0. He scores a plus point for every centimeter below the surface of the stool. Otherwise, he scores a minus point for every centimeter above the surface of the stool. The test is invalid if, during bending, the legs are bent in knee joints. Any vigorous movements during bends are not permitted, either. The result is the best of two attempts.

All measurements were taken in September 2019 (marking the beginning of academic year), and in January 2020 (20 weeks later, at the end of the first semester). The final, third examination took place in September 2020 (8 months after the end of the Brainball Intervention Program) to estimate the long-term impact. Technical researchers conducted fitness tests on the training ground during physical education classes. Measurements were carried out in a natural environment with the help of teachers and students. Additionally, principals, teachers, and parents approved information on testing procedures before testing.

### 2.3. Data analysis

Statistica software version 13.0 (Statsoft Polska Sp. z o.o., Krakow, Poland) was used for statistical analysis. The main dependent variables were the scores on the physical fitness test obtained by testing students in the control and experimental groups. First, the Shapiro-Wilk test confirmed the normal distribution of the physical fitness test. Then, the students' *t*-tests for the dependent variables were used to compare the differences in the mean parameters of the tests performed between the experimental and control groups. Next, to determine the statistically significant differences between the experimental and control groups after 1 year of study, an analysis of variance (ANOVA) was performed. Partial eta squared (η*p*^2^) was used to quantify the effect size (small effect = 0.01; medium effect = 0.06; large effect = 0.14) (34). Newmana-Keulsa's *post-hoc* test was used to confirm the importance of the differences between groups. Statistical significance was set at *p* < 0.05.

## 3. Results

Table 2 presents data on the physical fitness level of pupils before and after the experiment in each group.

**Table 2:** The mean and standard deviation (SD) of the fitness test obtained by pupils from the experimental and control groups in the pre, post, and follow-up tests.

**Variables**		**Evaluation stage**			
		**Pre-test**	**Post-test**	***p***	**Follow-up**	***p***
50-meter running (s)	C	13.22 ± 0.30	13.08 ± 0.32	ns	12.42 ± 0.25	0.000
	E	13.40 ± 0.30	13.20 ± 0.31	ns	11.91 ± 0.24	0.000
	*p*-value	ns	ns		ns	
Toe touch (cm)	C	5.33 ± 0.63	6.08 ± 0.49	ns	8.22 ± 0.48	0.000
	E	3.54 ± 0.61	5.38 ± 0.48	ns	8.31 ± 0.47	0.000
	*p*-value	0.036	ns		ns	
Standing long jump (cm)	C	100.12 ± 2.35	100.36 ± 2.60	ns	113.70 ± 167	0.000
	E	101.29 ± 2.29	105.48 ± 2.54	ns	123.69 ± 1.63	0.000
	*p*-value	ns	ns		0.018	
4 × 10 m sprint (s)	C	16.30 ± 0.35	15.77 ± 0.23	ns	14.89 ± 0.23	0.000
	E	16.41 ± 0.33	15.48 ± 0.23	ns	14.22 ± 0.22	0.000
	*p*-value	ns	ns		ns	
Hand strength (kg)	C	10.41 ± 0.54	10.48 ± 0.45	ns	11.70 ± 0.33	0.000
	E	9.30 ± 0.53	9.86 ± 0.44	ns	11.67 ± 0.33	0.000
	*p*-value	ns	ns		ns	
Bent arm hang (s)	C	2.17 ± 0.17	2.56 ± 0.14	ns	3.83 ± 0.12	0.000
	E	2.37 ± 0.17	2.84 ± 0.14	ns	3.97 ± 0.12	0.000
	*p*-value	ns	ns		ns	
Sit-ups (num.)	C	8.31 ± 0.69	10.17 ± 0.53	ns	17.34 ± 0.72	0.000
	E	9.04 ± 0.67	11.35 ± 0.51	ns	17.40 ± 0.70	0.000
	*p*-value	ns	ns		ns	

C, Control group; E, Experimental group; ns, lack of statistical differences at p < 0.05.

The results in Table 2 showed that there were no significant differences between the two groups (experimental and control groups) at the beginning of the experiment. Pupils of the experimental and control groups had no significant improvement in their physical fitness level after 20 weeks of the experiment (pre to post-test). However, the physical fitness level of pupils from the two groups improved significantly at the follow-up test, *p* < 0.001.

**Figure 1:**
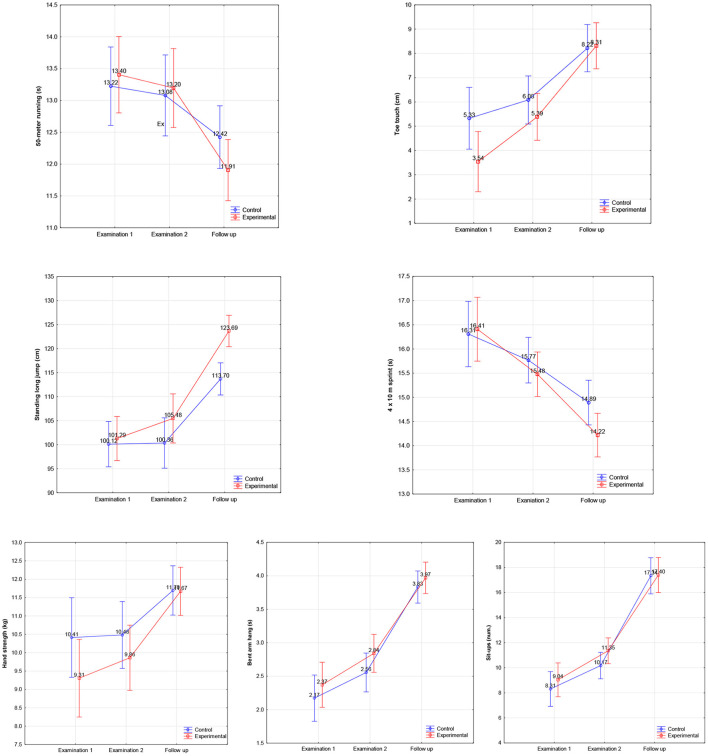
Physical fitness levels of pupils in the experimental and control groups at the first, second, and follow-up tests.

A repeated-measures analysis of variance ANOVA (2 × 2) was performed to compare the effectiveness of the Brainballs program on pupil physical fitness. The analysis showed that after 1 year of study, there were significant differences in the level of physical fitness improvement between the groups. Pupils in the experimental group were significantly better than those in the control group in most tests, especially the 50-meter run [*F*_(2, 102)_ = 5.72, *p* = 0.004] and toe touch [*F*_(2, 102)_ = 4.47, *p* = 0.014], standing long jump [*F*_(2, 102)_ = 5.11, *p* = 0.008], 4 × 10-meter sprint [*F*_(2, 102)_ = 3.52, *p* = 0.033], and hand strength [*F*_(2, 102)_ = 3.90, *p* = 0.023; Table 3, Figure 1].

**Table 3:** Analysis of variance for physical fitness levels by group condition.

**Variables**	**SS**	**MS**	***F***	***p***	***ηp* ^2^**
50-meter running (s)	3.98	1.99	5.72	0.004	0.101
Toe touch (cm)	23.86	11.93	4.47	0.014	0.081
Standing long jump (cm)	521	260	5.11	0.008	0.091
4 × 10 m sprint (s)	4.00	2.00	3.52	0.033	0.064
Hand strength (kg)	7.87	3.93	3.90	0.023	0.071
Bent arm hang (s)	0.15	0.08	0.38	ns	0.007
Sit-ups (num.)	8.54	4.27	0.59	ns	0.011

ns, lack of statistical difference at p < 0.05; SS, Sum of Squares; MS, Mean square; ηp^2^, partial eta square.

## 4. Discussion

The main objective of this study was to evaluate the effectiveness of Brainball program on the physical fitness of 7-year-old children in a primary school in Vietnam. The Brainball program is an innovative, comprehensive educational program that encourages and provides opportunities for intensive activities to improve health-related fitness and motor performance skills and enhance achievement learning to improve academic performance in comprehensive content. The study showed that after 20 weeks of the experiment, there was no significant difference in the level of physical development of the pupils in both groups (experimental and control groups). However, after a year of study, there were significant differences between the two groups. The improvement in physical fitness of the pupils in the experimental group was significantly better than that of the pupils in the control group, especially in 50-meter running (*p* = 0.004), toe touch (*p* = 0.014), standing long jump (*p* = 0.008), 4 × 10 m sprint (*p* = 0.033), and hand strength (*p* = 0.023) tests. These findings indicated that the Brainball program did not positively affect the pupils' physical fitness development in the experimental period, however, it showed progress in the follow-up, which may indicated long-term positive effects on the pupils' physical fitness development.

Previous studies found similar physical development results when using educational balls in PE classes for Polish preschool and primary school students (16, 18, 35, 36). Rokita (16) led a 3-year study (2004–2007) to assess the usefulness of the “Edubal” educational ball in engaging in physical activity combined with subject content (reading and writing). The study sample was students in grades 1–3 at two elementary schools. Each school has an experimental group and a control group. This is a longitudinal study with a total of six assessments of students' physical fitness (always at the beginning and end of each consecutive school year). The results of the study showed that the physical fitness development of the students was not affected by their participation in physical activities using the “Edubal” educational balls (16). Cichy and Rzepa (36) got similar results on the relationship between pupils' physical development and the use of educational ball in PE. Pupils participating in physical activities with educational balls do not adversely affect the change and development of their physical fitness. The authors concluded that lessons using educational balls could impact pupils' motor development as much as traditional lessons (36). Cichy (35) conducted a year-long study comparing the physical development, physical coordination, and learning abilities of first graders in traditional physical education classes compared to non-traditional classes. The findings suggest that a non-traditional curriculum using educational balls in physical education did not result in adverse changes in physical ability and general physical coordination but contributed to the more effective achievement of learning goals at this stage (35).

In this study, the application of the Brainball program to PE classes during the experiment did not affect the fitness of the pupils in the experimental group. One of the reasons can be provided by the relatively short practice time with Brainball (twice a week, 35 min each time). Compared with previous studies, when educational balls were applied in Poland (3 times a week, 45 min each time) in case of Vietnamese intervention the time duration (2 times a week 35 min) might have been an issue. Exciting physical activities with colorful balls (Brainball) could help pupils actively participate in movement, improve coordination, and develop motor skills, but did not impact level of fitness so significantly as the operational time was too short for pupils to absorb and develop fitness. Explanation may come from Rink (37), who states that fitness can only be developed when specific workload, movement duration and intensity standards are met (37).

The follow-up test results showed that after 1 year of study, the physical fitness of the pupils in the experimental group was significantly improved compared to the pupils in the control group, especially in terms of speed, strength, and flexibility. This suggests an association between the use of Brainballs in physical education classes and the physical development of pupils in the experimental group. Games and exercises with Brainball can be the cause of this relationship. Exciting activities with educational balls have made the class lively and attractive, students actively participate in and acquire knowledge (38). All these factors may help increase cognitive and emotional involvement in PE, which are the most important factors linked to further development and improvement of PA and physical fitness. The researchers also observed that the development of students' physical fitness and health was strongly related to improving the quality of physical education lessons. During the practice, attention should be paid to increasing the range of motion and intensity of movement for students (10, 39–41).

Researchers have previously demonstrated that the development of physical fitness is not only influenced by physical activities and sports, but also by factors such as genetics, environment (42); socioeconomic status of parents (43, 44); parental education (45); eating behavior (46). Therefore, with the present study's findings, it is impossible to conclude with certainty that the Brainball program has a positive long-term impact on the development of physical fitness for second-grade students in Vietnam. It is necessary to have further analyzes in the future about the aspects that can affect the physical development of students after the experimental period.

There are limitations to our study. Firstly, this is the first study to apply for the Brainballs program in physical education classes for students in Vietnam. Teachers and students are very interested in this new method of teaching and learning, but sometimes they are confused and unfamiliar with how to organize and perform exercises. Secondly, the study sample is relatively small, so it is difficult to generalize the research results. Despite its limitations, the study has many advantages. The study used an experimental pedagogical design with two parallel groups conducted in a natural environment. The students voluntarily participated in this study and received special attention from principals, teachers, and parents. During the implementation, classroom and physical education teachers coordinated rhythmically in the content of the curriculum, which is a new thing in the Vietnamese education system. The findings provide further insight into the effectiveness of using educational balls to promote healthy physical development in Vietnamese 7-year-olds' physical education classes. As for the pupils, they had the opportunity to experience new ways of learning and participate in exciting sports activities with Brainballs. This helped increase their interest while learning and fostering the absorption of knowledge. In addition, the results of this study will be the premise for further studies with a broader and deeper scope of research to precisely evaluate the impact of Brainball on the physical fitness development of students.

## Data availability statement

The raw data supporting the conclusions of this article will be made available by the authors, without undue reservation.

## Ethics statement

The studies involving human participants were reviewed and approved by Resolution of the Senate Committee on Ethics of Scientific Research at the Wroclaw University of Health and Sport Sciences in Wroclaw. Written informed consent to participate in this study was provided by the participants' legal guardian/next of kin.

## Author contributions

VP, AR, and MB developed ideas, managed the project, provided critical scholarly guidance for the drafting and interpretation of the manuscript, and critically revised the intellectual content of the manuscript. VP, SW, IC, and MB researched background literature, analyzed data, and wrote the manuscript. All authors approved the final version of the manuscript, ensured the accuracy of the work, and agreed to take responsibility for all aspects of the work.
